# TGF-β1 Up-Regulates Connective Tissue Growth Factor Expression in Human Granulosa Cells through Smad and ERK1/2 Signaling Pathways

**DOI:** 10.1371/journal.pone.0126532

**Published:** 2015-05-08

**Authors:** Jung-Chien Cheng, Hsun-Ming Chang, Lanlan Fang, Ying-Pu Sun, Peter C. K. Leung

**Affiliations:** 1 Department of Obstetrics and Gynaecology, Child & Family Research Institute, University of British Columbia, Vancouver, British Columbia, V5Z 4H4, Canada; 2 Reproductive Medical Center, The First Affiliated Hospital of Zhengzhou University, Zhengzhou, 450052, China; University of Quebec at Trois-Rivieres, CANADA

## Abstract

Connective tissue growth factor (CTGF), which is also called CCN2, is a secreted matricellular protein. CTGF regulates various important cellular functions by interacting with multiple molecules in the microenvironment. In the ovary, CTGF is mainly expressed in granulosa cells and involved in the regulation of follicular development, ovulation and luteinization. TGF-β1 has been shown to up-regulate CTGF expression in rat and hen granulosa cells. However, the underlying molecular mechanisms of this up-regulation remain undefined. More importantly, whether the stimulatory effect of TGF-β1 on CTGF expression can be observed in human granulosa cells remains unknown. In the present study, our results demonstrated that TGF-β1 treatment up-regulates CTGF expression in both immortalized human granulosa cells and primary human granulosa cells. Using a siRNA-mediated knockdown approach and a pharmacological inhibitor, we demonstrated that the inhibition of Smad2, Smad3 or ERK1/2 attenuates the TGF-β1-induced up-regulation of CTGF. This study provides important insights into the molecular mechanisms that mediate TGF-β1-up-regulated CTGF expression in human granulosa cells.

## Introduction

Connective tissue growth factor (CTGF) is a secreted protein that belongs to the CCN family, which consists of the following six members: cysteine-rich protein 61 (CYR61/CCN1), CTGF (CCN2), nephroblastoma overexpressed gene (NOV/CCN3), Wnt-inducible secreted protein-1 (WISP-1/CCN4), WISP-2 (CCN5) and WISP-3 (CCN6) [1]. The CCN family of proteins, which are known as matricellular proteins, primarily act as modulators that regulate multiple cellular functions in response to various environmental stimuli [2]. Thus far, CCN family members have been shown to regulate cell proliferation, apoptosis, migration, differentiation and extracellular matrix remodeling [3]. CTGF, which was originally identified as a growth factor-inducible immediate early gene in mouse fibroblasts and in human vascular endothelial cells, is the most studied member of this family [4, 5]. CTGF is expressed in multiple cell types and plays important regulatory roles in female reproductive organs [6, 7].

Animal studies have shown that CTGF is expressed in granulosa cells and that CTGF expression levels increase during follicular development [8, 9]. Compared to granulosa cells, CTGF mRNA is expressed at low abundance in theca cells. Thus, ovarian CTGF is mainly produced by granulosa cells [10]. Interestingly, CTGF mRNA levels are down-regulated in the granulosa cells of preovulatory follicles but are up-regulated again after ovulation [9, 10]. Importantly, knockout mouse studies have shown that the conditional knockout of CTGF in the ovary and uterus results in reduced fertility, disrupted follicular development, decreased ovulation and enhanced corpus luteum formation [11]. Moreover, many studies have suggested that granulosa cell-derived CTGF likely plays a critical role in the regulation of theca cell recruitment, follicle growth and corpus luteum vascularization [8–10, 12]. Taken together, these results clearly indicate that CTGF acts as an autocrine/paracrine factor to regulate follicular development, ovulation and luteinization [11, 13].

Many animal studies have shown that transforming growth factor-beta 1 (TGF-β1) can regulate ovarian steroidogenesis, granulosa cell proliferation and differentiation [14, 15]. In humans, TGF-β1 protein can be detected in the follicular fluid [16, 17]. Additionally, TGF-β1 and its receptors, TGF-β receptor type I (TβRI) and type II (TβRII) are all expressed in granulosa cells [18–20]. However, although the expression of TGF-βs and TGF-β receptors have been identified in the human ovary, to date, only a handful of studies have investigated the functions of TGF-β1 in human granulosa cells. TGF-β1 exerts its functions by activating canonical and non-canonical signaling pathways. In the canonical pathway, the downstream signaling molecules Smad2 and Smad3 are phosphorylated and activated upon ligand binding to the receptor, and in combination with common Smad4, subsequently translocate into the nucleus where these molecules mediate TGF-β 1-regulated gene expression [21]. The non-canonical signaling pathway, which is also called the non-Smad signaling pathway, is involved the activation of MAPK, PI3K/Akt and Rho GTPase signaling pathways [22].

In other cell types, CTGF is regulated by various growth factors, cytokines and hormones [6]. In rat granulosa cells, CTGF mRNA levels are up-regulated by estrogen and by 5α-dihydrotestosterone (DHT) but down-regulated by follicle stimulating hormone (FSH) and by human chorionic gonadotropin (hCG) [8, 10, 12]. We recently demonstrated that treatment of TGF-β 1 up-regulates cyclooxygenase-2 (COX-2) expression and increases prostaglandin E2 (PGE2) production but down-regulates StAR expression and decreases progesterone production in human granulosa cells. These previous findings confirmed the functional roles of TGF-β 1 in human granulosa cells, particularly in regulating ovulation and luteinization [23, 24]. In rat granulosa cells, TGF-β 1 has been shown to up-regulate CTGF expression [12]. In addition, the expression levels of CTGF in hen granulosa cells are up-regulated by TGF-β1 and the stimulatory effect of TGF-β1 is blocked by treatment with the TβRI inhibitor SB431542 [25]. However, whether the same mechanism is true for human granulosa cells remains unknown. Given the important roles of CTGF in granulosa functions, the present study was designed to investigate the effect of TGF-β 1 on CTGF expression and to determine the underlying molecular mechanisms in human granulosa cells.

## Materials and Methods

### Cell culture

A non-tumorigenic SV40 large T antigen immortalized human granulosa cell line (SVOG) that was previously established by our group was used in the present study [26]. SVOG cells retain the steroidogenic activities, including basal as well as 8-Br-cAMP- or human chorionic gonadotropin (hCG)-stimulated progesterone secretion [26]. Moreover, we have shown that several members of TGF-β superfamily can regulate the StAR expression and progesterone production in SVOG cells [24, 27, 28]. However, compared to those of primary human granulosa cells, the expression levels of cytochrome P450 aromatase and FSH receptor were relatively low in SVOG cells [24]. The cells were grown in DMEM/F12 medium (Sigma-Aldrich, Oakville, ON) and supplemented with 10% charcoal/dextran-treated fetal bovine serum (Hyclone Laboratories Inc., Logan, UT). The cultures were maintained at 37°C in a humidified atmosphere of 5% CO_2_.

### Preparation of primary human granulosa cells

Primary human granulosa cells were obtained with informed patient consent following approval from the University of British Columbia Research Ethics Board. The controlled ovarian stimulation protocol for *in vitro* fertilization patients consisted of either luteal-phase naferelin acetate (Synarel, Pfizer, Kirkland, Quebec, Canada) or follicular phase GnRH antagonist (Ganirelix; Merck Canada) down-regulation. Gonadotropin stimulation began on menstrual cycle day 2 with human menopausal gonadotropin (hMG; Menopur, Ferring, Canada) and recombinant FSH (Puregon, Merck, Canada) and was followed by human chorionic gonadotropin administration 34–36 hours before oocyte retrieval, based on follicle size. Granulosa cells were purified by density centrifugation from follicular aspirates collected from women undergoing oocyte retrieval as previously described [29, 30].

### Antibodies and reagents

Polyclonal anti-CTGF (diluted at 1:1000) and monoclonal anti-α-tubulin (diluted at 1:5000) antibodies were obtained from Santa Cruz Biotechnology (Santa Cruz, CA). Polyclonal anti-Smad4 (diluted at 1:1000), anti-TGF-β receptor type I (diluted at 1:1000), anti-ERK1/2 (diluted at 1:2000), monoclonal anti-Smad2 (diluted at 1:1000) and anti-Smad3 (diluted at 1:1000) antibodies were obtained from Cell Signaling Technology (Danvers, MA). Horseradish peroxidase-conjugated goat anti-mouse and goat anti-rabbit IgG (diluted at 1:5000) were obtained from Bio-Rad Laboratories (Hercules, CA), and horseradish peroxidase-conjugated donkey anti-goat IgG (diluted at 1:5000) was obtained from Santa Cruz Biotechnology. Recombinant human TGF-β 1 was obtained from R&D systems (Minneapolis, MN). SB431542 was obtained from Sigma-Aldrich (Oakville, ON), and U0126 was obtained from Calbiochem (San Diego, CA).

### Reverse transcription quantitative real-time PCR (RT-qPCR)

Total RNA was extracted using TRIzol reagent (Invitrogen, Life Technologies, Burlington, ON) according to the manufacturer’s instructions. Reverse transcription was performed with 3 μg of RNA, random primers and M-MLV reverse transcriptase (Promega, Madison, WI). The primers were designed using Primer Express Software v2.0 (Applied Biosystems, Foster City, CA). All primers spanned at least one intron to detect specific mRNA sequences. The following primers were used for SYBR Green reverse transcription-qPCR (RT-qPCR): CTGF, 5'-GCG TGT GCA CCG CCA AAG AT-3' (sense) and 5'-CAG GGC TGG GCA GAC GAA CG-3' (antisense); TGF-β receptor type I, 5'-GTT AAG GCC AAA TAT CCC AAA CA-3' (sense) and 5'-ATA ATT TTA GCC ATT ACT CTC AAG G-3' (antisense); Smad2, 5'-GCC TTT ACA GCT TCT CTG AAC AA-3' (sense) and 5'-ATG TGG CAA TCC TTT TCG AT-3' (antisense); Smad3, 5'-CCC CAG CAC ATA ATA ACT TGG-3' (sense) and 5'-AGG AGA TGG AGC ACC AGA AG-3' (antisense); Smad4, 5'-TGG CCC AGG ATC AGT AGG T-3' (sense) and 5'-CAT CAA CAC CAA TTC CAG CA-3' (antisense) and GAPDH, 5'-GAG TCA ACG GAT TTG GTC GT-3' (sense) and 5'-GAC AAG CTT CCC GTT CTC AG-3' (antisense). RT-qPCR was performed using an Applied Biosystems 7300 Real-Time PCR System equipped with a 96-well optical reaction plate. The specificity of each assay was validated by melting curve analysis and by agarose gel electrophoresis of the PCR products. All of the RT-qPCR experiments were run in triplicate, and a mean value was used to determine the mRNA levels. Water and mRNA without RT were used as negative controls. Relative quantification of the mRNA levels was performed using the comparative Ct method with GAPDH as the reference gene and using the formula 2^–∆∆Ct^. In RT-qPCR, to make the ∆∆Ct calculation be valid, the amplification efficiencies of the target and reference must be approximately equal. A sensitive method for assessing if two amplicons have the same efficiency is to look at how ∆Ct varies with template dilution [31]. Based on the Applied Biosystems Real-Time PCR Systems Guideline (Part Number 4348358), the absolute value of the slope of log input amount versus ∆Ct should be less than 0.1. All primers used in this study passed the validation test.

### Western blot analysis

Following treatment, cells were washed with cold PBS and lysed in lysis buffer (Cell Signaling) containing protease inhibitor cocktail (Sigma-Aldrich). Extracts were centrifuged at 20,000x *g* for 10 min at 4°C to remove cellular debris, and protein concentrations were quantified using the DC Protein Assay (Bio-Rad Laboratories). Equal amounts (50 μg) of protein were separated by SDS polyacrylamide gel electrophoresis and transferred onto PVDF membranes. After being blocked for 1 hour with 5% non-fat dry milk in Tris-buffered saline (TBS), the membranes were incubated overnight at 4°C with primary antibodies that were diluted in 5% non-fat milk-TBS. Following primary antibody incubation, the membranes were incubated with the appropriate HRP-conjugated secondary antibody. Immunoreactive bands were detected using an enhanced chemiluminescent substrate and X-ray film. The intensities of the bands were quantified by densitometric analysis using Scion Image software (Scion Corp, Frederick, MD).

### Small interfering RNA (siRNA) transfection

To knock down endogenous TGF-β receptor type I, Smad4, Smad2, Smad3 or ERK1/2, cells were transfected with 50 nM ON-TARGET*plus* SMART*pool* TGF-β receptor I, Smad4, Smad2, Smad3 or ERK1/2 siRNA (Dharmacon, Lafayette, CO) using Lipofectamine RNAiMAX (Invitrogen, Life Technologies). siCONTROL NON-TARGETING *pool* siRNA (Dharmacon) was used as the transfection control.

### Statistical analysis

The results are presented as the mean ± SEM of at least three independent experiments. For experiments involving only two groups, the data were analyzed by Excel with a Two-Sample *t*-test assuming unequal variances. Multiple group comparisons were analyzed by one-way ANOVA followed by Tukey’s multiple comparison test using PRISM software. Normality tests have been tested by D'Agostino-Pearson normality test. A significant difference was defined as *p*<0.05.

## Results

### TGF-β1 up-regulates CTGF expression in human granulosa cells

**Fig 1:**
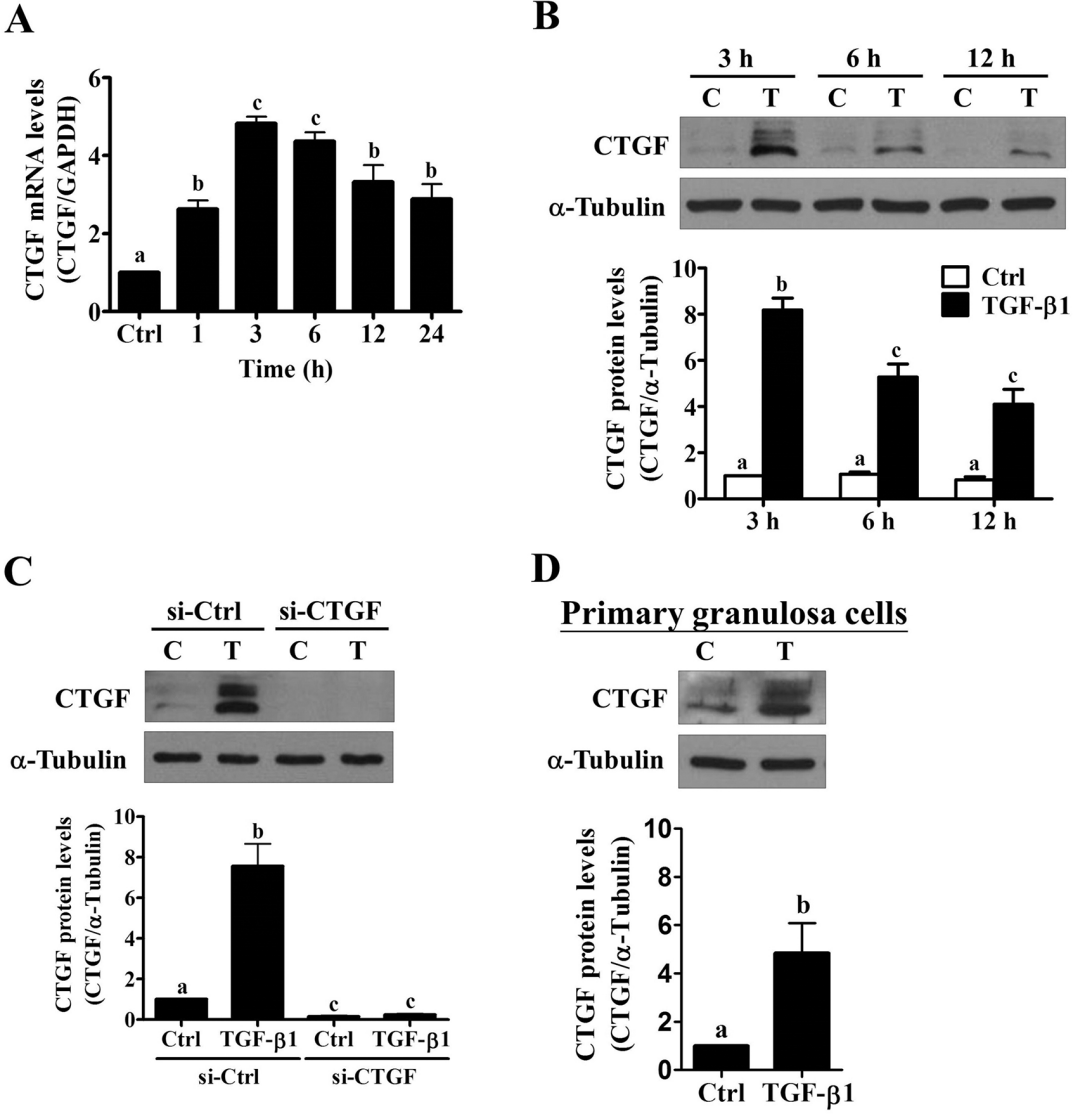
TGF-β1 up-regulates CTGF expression in human granulosa cells. (A) SVOG cells were treated with 5 ng/mL TGF-β1, and the mRNA levels of CTGF were analyzed at different time points by RT-qPCR. The level of CTGF mRNA at each time point was normalized to the GAPDH mRNA level at the same time point. (B) SVOG cells were treated with 5 ng/mL TGF-β1 for 3, 6 and 12 hours, and the protein levels of CTGF were examined by western blot. The level of CTGF protein at each time point was normalized to the β-tubulin protein level of 3 hour control. (C) SVOG cells were transfected for 48 hours with 50 nM control siRNA (si-Ctrl) or CTGF siRNA (si-CTGF) and then treated for 3 hours with 5 ng/mL TGF-β1. The protein levels of CTGF were examined by western blot. (D) Primary human granulosa cells were treated with 5 ng/mL TGF-β1 for 3 hours, and the protein levels of CTGF were examined by western blot. The results are expressed as the mean ± SEM of at least three independent experiments. Values without a common letter were significantly different (*p*<0.05).

Our previously established SVOG cells were used to examine the effect of TGF-β1 on CTGF expression in human granulosa cells [26]. As shown in Fig 1A, treatment with 5 ng/mL TGF-β1 significantly up-regulated CTGF mRNA levels after 1 hour. The most significant effect was observed after 3 hours of TGF-β1 treatment and then decreased but remained detectable after 24 hours of treatment. The western blot results showed that SVOG cells expressed CTGF and that the protein levels were up-regulated after 3, 6 and 12 hours of TGF-β1 treatment. Similar to the RT-qPCR results, the maximal stimulatory effect of TGF-β1 on CTGF protein levels was observed after 3 hours of treatment (Fig 1B). CTGF can be glycosylated, which results in different protein sizes [32]. Thus, the glycosylation of CTGF could explain the multiple bands that were observed in our western blot results. A siRNA-mediated knockdown approach was used to knockdown CTGF expression to confirm the specificity of the CTGF antibody. As shown in Fig 1C, the transfection of SVOG cells with CTGF siRNA down-regulated the basal levels of CTGF expression. Moreover, the TGF-β1-induced up-regulation of CTGF was abolished by CTGF knockdown. Notably, all bands were affected by the TGF-β1 and CTGF siRNA treatments, which confirmed the specificity of the CTGF antibody that was used in the present study. To further confirm the stimulatory effect of TGF-β1 on CTGF expression in human granulosa cells, primary human granulosa cells obtained from patients undergoing *in vitro* fertilization procedure were treated with 5 ng/mL TGF-β1 for 3 hours. As our western results shown in Fig 1D, consistent with our findings in SVOG cells, treatment with TGF-β1 significantly up-regulated CTGF protein levels in primary human granulosa cells.

### TGF-β receptor is required for the TGF-β1-induced up-regulation of CTGF expression

**Fig 2:**
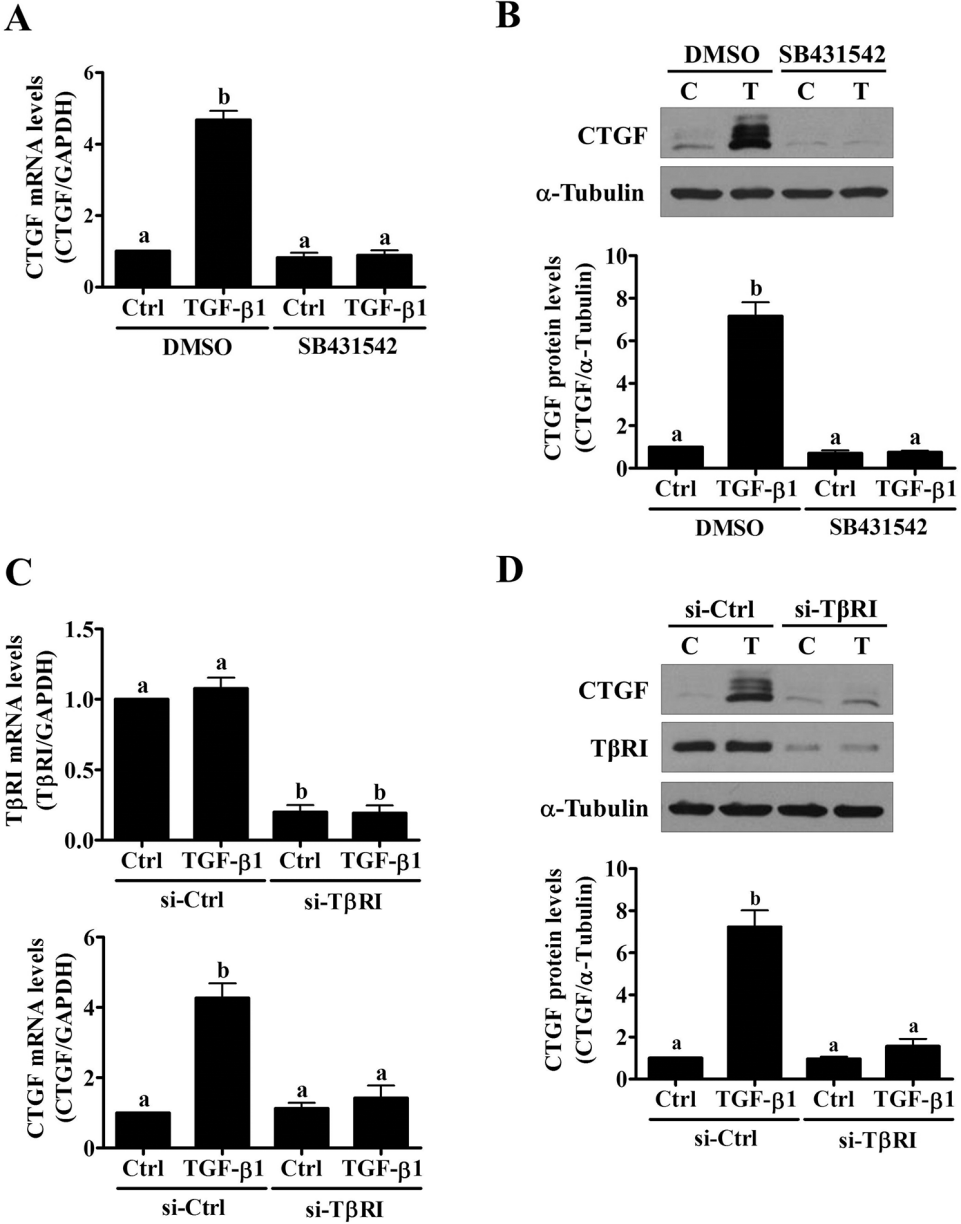
The TGF-β receptor is required for the TGF-β1-induced up-regulation of CTGF expression in SVOG cells. (A) and (B) SVOG cells were pretreated with 10 μM SB431542 for 1 hour and then treated with 5 ng/mL TGF-β1 for 3 hours. The mRNA (A) and protein (B) levels of CTGF were examined by RT-qPCR and western blot, respectively. (C) and (D) SVOG cells were transfected with 50 nM control siRNA (si-Ctrl) or TβRI siRNA (si-TβRI) for 48 hours and then treated with 5 ng/mL TGF-β1 for 3 hours. The mRNA (C) and protein (D) levels of CTGF and TβRI were examined by RT-qPCR and western blot, respectively. The results are expressed as the mean ± SEM of at least three independent experiments. Values without a common letter were significantly different (*p*<0.05).

A potent and specific TGF-β1 type I receptor (TβRI) inhibitor, SB431542, was used to block the activation of TβRI to confirm the requirement of this receptor for the TGF-β1-induced up-regulation of CTGF expression [33]. As shown in Fig 2A, treatment with 10 μM SB431542 did not affect the basal mRNA levels of CTGF but abolished the TGF-β1-induced up-regulation of CTGF mRNA levels. The inhibitory effect of SB431542 on the TGF-β1-induced up-regulation of CTGF protein levels was confirmed by western blot analyses (Fig 2B). Then, a siRNA-mediated knockdown approach was used to avoid the possible off-target effects of SB431542. As shown in Fig 2C and 2D, the transfection of TβRI siRNA significantly down-regulated TβRI mRNA and protein levels in SVOG cells. In addition, the TGF-β1-up-regulated CTGF mRNA and protein levels were abolished by TβRI knockdown.

### Smad2 and Smad3 are involved in the TGF-β1-induced up-regulation of CTGF expression

**Fig 3:**
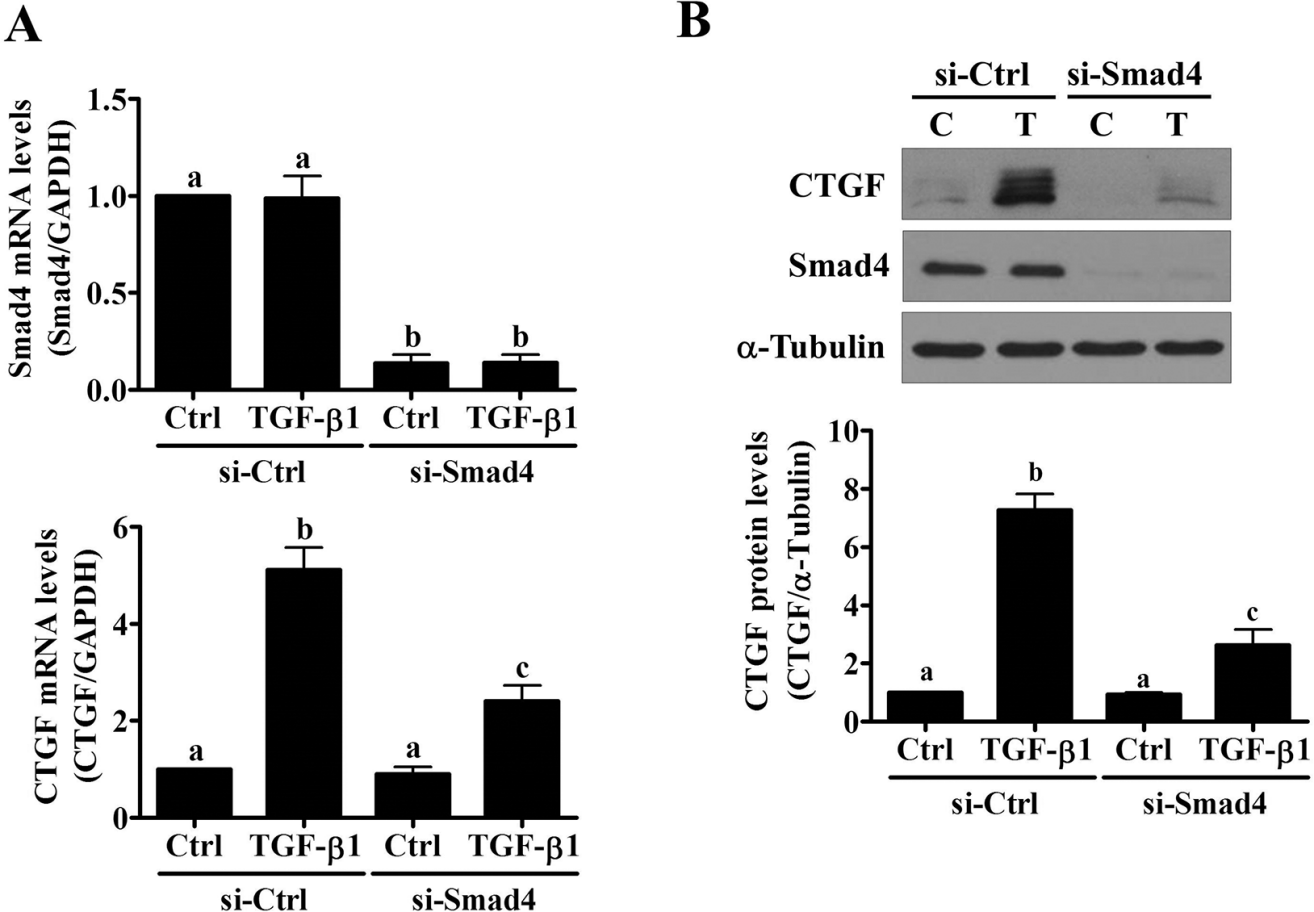
Smad signaling pathway is required for the TGF-β1-induced up-regulation of CTGF expression in SVOG cells. (A) and (B) SVOG cells were transfected with 50 nM control siRNA (si-Ctrl) or Smad4 siRNA (si-Smad4) for 48 hours and then treated with 5 ng/mL TGF-β1 for 3 hours. The mRNA (A) and protein (B) levels of CTGF and Smad4 were examined by RT-qPCR and western blot, respectively. The results are expressed as the mean ± SEM of at least three independent experiments. Values without a common letter were significantly different (*p*<0.05).

**Fig 4:**
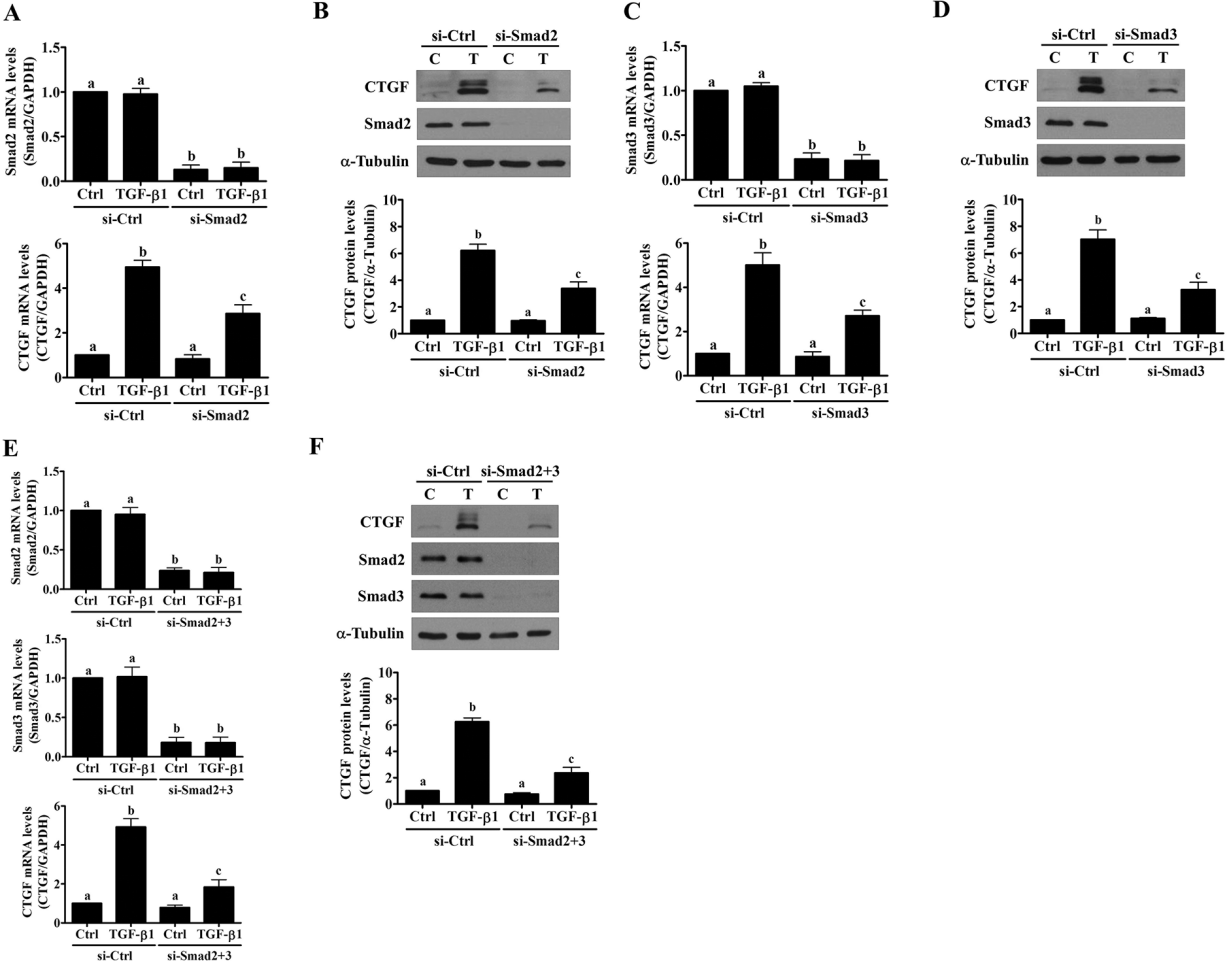
Knockdown of Smad2 or Smad3 attenuates TGF-β1-induced up-regulation of CTGF expression in SVOG cells. (A) and (B) SVOG cells were transfected with 50 nM control siRNA (si-Ctrl) or Smad2 siRNA (si-Smad2) for 48 hours and then treated with 5 ng/mL TGF-β1 for 3 hours. The mRNA (A) and protein (B) levels of CTGF and Smad2 were examined by RT-qPCR and western blot, respectively. (C) and (D) SVOG cells were transfected with 50 nM control siRNA (si-Ctrl) or Smad3 siRNA (si-Smad3) for 48 hours and then treated with 5 ng/mL TGF-β1 for 3 hours. The mRNA (C) and protein (D) levels of CTGF and Smad3 were examined by RT-qPCR and western blot, respectively. (E) and (F) SVOG cells were transfected with 50 nM control siRNA (si-Ctrl) or Smad2 plus Smad3 siRNAs (si-Smad2+3) for 48 hours and then treated with 5 ng/mL TGF-β1 for 3 hours. The mRNA (E) and protein (F) levels of CTGF, Smad2 and Smad3 were examined by RT-qPCR and western blot, respectively. The results are expressed as the mean ± SEM of at least three independent experiments. Values without a common letter were significantly different (*p*<0.05).

Common Smad4 siRNA was used to block the transduction of Smad signaling to examine the involvement of Smad signaling pathways in the TGF-β1-induced up-regulation of CTGF expression. As shown in Fig 3A and 3B, Smad4 knockdown partially attenuated the TGF-β1-induced up-regulation of CTGF mRNA and protein levels. Smad2 and Smad3 can mediate TGF-β1-regulated gene expression redundantly and differentially in a context-dependent manner [34]. Therefore, Smad2 or Smad3 expression was knocked down using a specific siRNA to determine which Smad was required for the TGF-β1-induced up-regulation of CTGF expression. As shown in Fig 4A and 4B, the transfection of Smad2 siRNA significantly down-regulated endogenous Smad2 mRNA and protein levels. In addition, Smad2 knockdown partially attenuated the TGF-β1-induced up-regulation of CTGF mRNA and protein levels (Fig 4A and 4B). Similarly, TGF-β1-up-regulated CTGF mRNA and protein levels were partially attenuated by Smad3 knockdown (Fig 4C and 4D). Combined knockdown of Smad2 and Smad3 resulted in further attenuation of TGF-β1-induced up-regulation of CTGF mRNA and protein levels, which were similar to the results obtained from Smad4 knockdown (Fig 4E and 4F).

### The ERK1/2 signaling pathway is involved in the TGF-β1-induced up-regulation of CTGF expression

**Fig 5:**
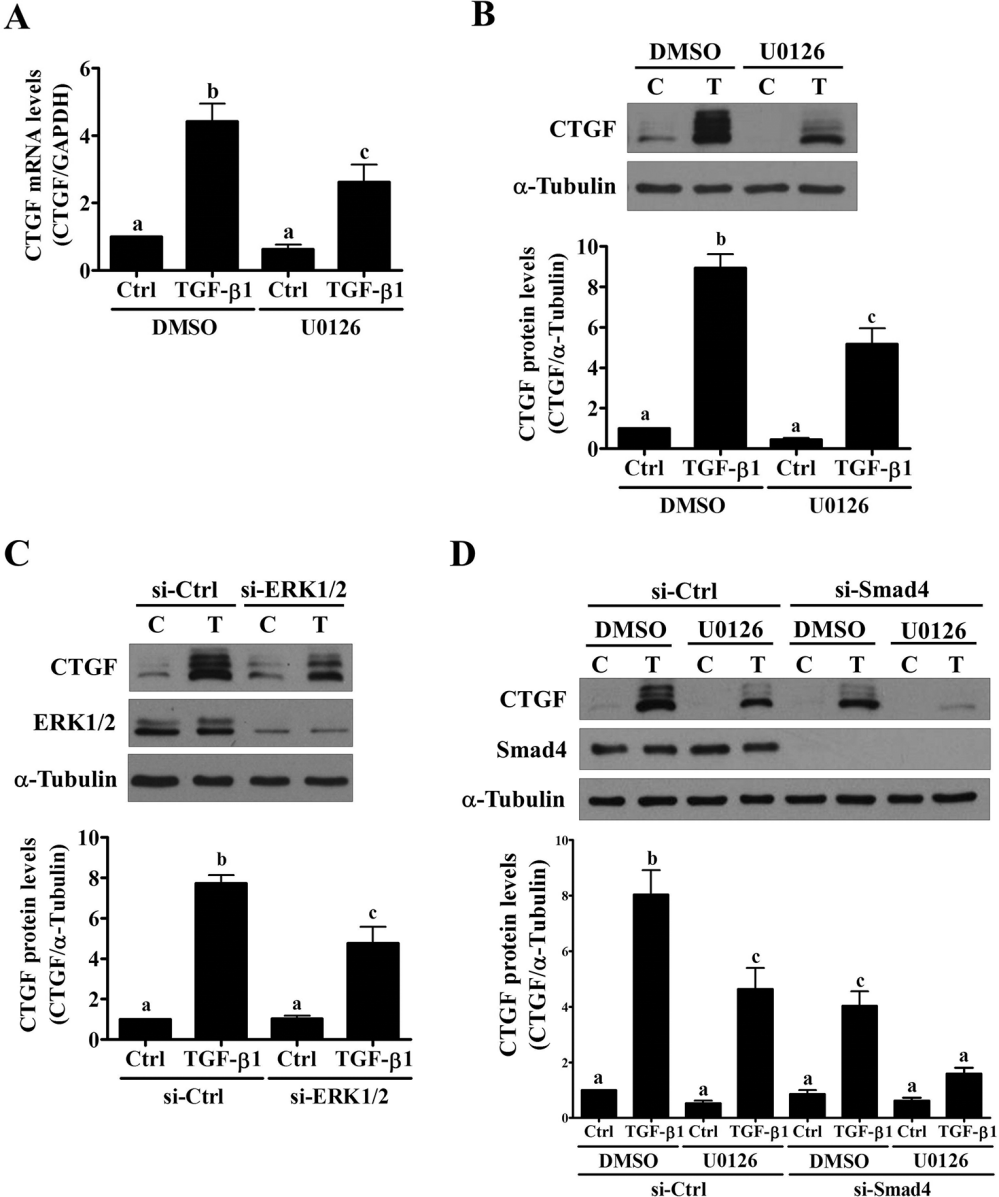
The ERK1/2 signaling pathway is required for the TGF-β1-induced up-regulation of CTGF expression in SVOG cells. (A) and (B) SVOG cells were pretreated with 10 μM U0126 for 1 hour and then treated with 5 ng/mL TGF-β1 for 3 hours. The mRNA (A) and protein (B) levels of CTGF were examined by RT-qPCR and western blot, respectively. (C) SVOG cells were transfected with 50 nM control siRNA (si-Ctrl) or ERK1/2 siRNAs (si-ERK1/2) for 48 hours and then treated with 5 ng/mL TGF-β1 for 3 hours. The protein levels of CTGF and ERK1/2 were examined by western blot. (D) SVOG cells were transfected with 50 nM control siRNA (si-Ctrl) or Smad4 siRNA (si-Smad4) for 48 hours and then pretreated with U0126 (10 μM) for 1 hour. After pretreatment, the cells were treated with 5 ng/mL TGF-β1 for 3 hours. The protein levels of CTGF were examined by western blot. The results are expressed as the mean ± SEM of at least three independent experiments. Values without a common letter were significantly different (*p*<0.05).

We focused on Smad-independent signaling pathways to examine other possible signaling pathways that are involved in TGF-β1-up-regulated CTGF expression. Our previous study have demonstrated that TGF-β1 can activate the ERK1/2, but not PI3K/Akt, signaling pathway in SVOG cells [24]. Therefore, the MEK inhibitor U0126 was used to block the activation of ERK1/2. As shown in Fig 5A and 5B, U0126 treatment partially attenuated the TGF-β1-induced up-regulation of CTGF mRNA and protein levels. To avoid the off-target effects of the pharmacological inhibitor and to further confirm the requirement of the ERK1/2 signaling in the TGF-β1-induced CTGF up-regulation, ERK1/2 siRNAs were used to knock down endogenous ERK1/2 expression. As shown in Fig 5C, transfection with ERK1/2 siRNAs significantly down-regulated ERK1/2 protein levels. Moreover, knockdown of ERK1/2 attenuated the TGF-β1-induced up-regulation of CTGF protein levels. Interestingly, the inhibition of Smad and ERK1/2 simultaneously by transfecting the cells with Smad4 siRNA and by treating the cells with U0126 resulted in further attenuation of the TGF-β1-induced up-regulation of CTGF protein levels (Fig 5D). These results indicated that both Smad and ERK1/2 signaling pathways were required for the TGF-β1-induced up-regulation of CTGF in human granulosa cells.

## Discussion

Thus far, only few studies have investigated the regulation of CTGF in granulosa cells of different animal models; additionally, the underlying molecular mechanisms of this regulation are poorly understood. More importantly, the regulation of CTGF expression in human granulosa cells is largely unknown. In the present study, our results showed that CTGF were expressed in SVOG and primary human granulosa cells. These results are consistent with the results of previous studies that demonstrated that CTGF is expressed in human granulosa cells [35, 36]. In addition, using SVOG cells as our experimental model, this study is the first to demonstrate that the expression of CTGF expression in human granulosa cells was up-regulated by TGF-β1 through the activation of Smad and ERK1/2 signaling pathways in human granulosa cells. In rat granulosa cells, CTGF mRNA levels are up-regulated by theca cell-, granulosa cell- and oocyte-derived TGF-β superfamily members that include TGF-β1, activin A and growth differentiation factor 9. Interestingly, the stimulatory effects of these TGF-β superfamily members are abolished by the co-treatment with FSH. These results indicate that the regulation of CTGF expression in granulosa cells is multifactorial and acts in an autocrine/paracrine manner [12]. However, whether the same regulatory mechanism is true for human granulosa cells remains unknown. Therefore, future studies will be needed to examine the effects of gonadotropins or other local ovarian factors on TGF-β1-induced up-regulation of CTGF in human granulosa cells.

A recent review article summarized the transcriptional and post-transcriptional regulation of CTGF expression. Many transcription factors and microRNAs are involved with the various growth factors, cytokines and hormones that regulate CTGF expression [37]. Among these factors, TGF-β1 is the most profound factor that can up-regulate CTGF expression in many different cell types. A Smad binding site has been identified in the CTGF promoter in mouse fibroblast cells. In addition, Smad3, but not Smad2, is required for the TGF-β1-induced up-regulation of CTGF expression [38]. Similarly, the requirement of Smad3, but not Smad2, for the TGF-β1-induced up-regulation of CTGF expression has been shown in rat nucleus pulposus cells and hepatic stellate cells [39, 40]. Interestingly, TGF-β1-up-regulated CTGF expression is not mediated by the Smad signaling pathway in rat hepatic progenitor cells [41]. In human aortic smooth muscle cells, Runx2 represses TGF-β1-up-regulated CTGF expression by interacting with Smad3, which prevents the binding between Smad3 and the Smad-binding element [42]. Notably, little is known regarding the direct involvement of Smad2 or Smad3 in the TGF-β1-induced up-regulation of CTGF expression in any type of human cells thus far. In the present study, we applied a siRNA-mediated knockdown approach, and our results showed that the inhibition of both Smad2 and Smad3 attenuated TGF-β1-up-regulated CTGF expression in SVOG cells. Our results clearly showed that Smad2 and Smad3 are required for the TGF-β1-induced up-regulation of CTGF expression in human granulosa cells. Taken together, these results indicate that the regulation of CTGF by TGF-β1 is not only species-dependent but also cell type-dependent.

In the present study, our results showed that the inhibition of Smad signaling did not block the TGF-β1-induced up-regulation of CTGF expression completely, suggesting that additional factors were involved and that the regulation of CTGF expression by TGF-β1 was multifactorial. Indeed, various signaling pathways, including ERK1/2, p38 MAPK, JNK, STAT3 and PKC, are involved in the TGF-β1-induced up-regulation of CTGF expression in other cell types [40, 41, 43, 44]. In the present study, the inhibition of ERK1/2 signaling only partially attenuated the stimulatory effect of TGF-β1 on CTGF expression, which indicated that ERK1/2 was also involved in the TGF-β1-induced up-regulation of CTGF expression in human granulosa cells. Interestingly, the simultaneously inhibition of the Smad and ERK1/2 signaling pathways resulted in an increase in the attenuation of the TGF-β1-induced up-regulation of CTGF expression but could not completely inhibit this up-regulation. These results indicated that other signaling pathways in addition to Smad and ERK1/2 were involved in TGF-β1-up-regulated CTGF expression in SVOG cells. We demonstrated that the PI3K/Akt signaling pathway is not activated by TGF-β1 in SVOG cells [24]. However, whether other non-Smad signaling pathways can be activated by TGF-β1 and mediate the TGF-β1-induced up-regulation of CTGF expression in SVOG cells is unknown and warrants further investigation.

In summary, the present study demonstrates the stimulatory effect of TGF-β1 on CTGF expression in human granulosa cells. In addition, our results indicated that Smad2-, Smad3- and ERK1/2-mediated signaling pathways are involved in the TGF-β1-induced up-regulation of CTGF expression in human granulosa cells. These results provide not only important insights into the molecular mechanisms that mediate TGF-β1-up-regulated CTGF expression in human granulosa cells but also increase the understanding of the normal physiological roles of TGF-β1 in the ovary.
